# Prognostic analysis of hepatocellular carcinoma based on cuproptosis -associated lncRNAs

**DOI:** 10.1186/s12876-024-03219-6

**Published:** 2024-04-23

**Authors:** Mingwei Wei, Libai Lu, Zongjiang Luo, Jiasheng Ma, Jianchu Wang

**Affiliations:** 1 Guangxi Clinical Medical Research Center for Hepatobiliary Diseases, The Affiliated Hospital of Youjiang Medical University for Nationalities, Baise, China; 2 Department of Hepatobiliary and Pancreatic Surgery, Baidong Hospital, The Affiliated Hospital of Youjiang Medical University for Nationalities, Baise, China

**Keywords:** Cuproptosis, lncRNA, HCC, Genomic, Immune

## Abstract

**Objectives:**

Cuproptosis represents an innovative type of cell death, distinct from apoptosis, driven by copper dependency, yet the involvement of copper apoptosis-associated long non-coding RNAs (CRLncRNAs) in hepatocellular carcinoma (HCC) remains unclear. This study is dedicated to unveiling the role and significance of these copper apoptosis-related lncRNAs within the context of HCC, focusing on their impact on both the development of the disease and its prognosis.

**Methods:**

We conducted an analysis of gene transcriptomic and clinical data for HCC cases by sourcing information from The Cancer Genome Atlas database. By incorporating cuproptosis-related genes, we established prognostic features associated with cuproptosis-related lncRNAs. Furthermore, we elucidated the mechanism of cuproptosis-related lncRNAs in the prognosis and treatment of HCC through comprehensive approaches, including Lasso and Cox regression analyses, survival analyses of samples, as well as examinations of tumor mutation burden and immune function.

**Results:**

We developed a prognostic model featuring six cuproptosis-related lncRNAs: AC026412.3, AC125437.1, AL353572.4, MKLN1-AS, TMCC1-AS1, and SLC6A1-AS1. This model demonstrated exceptional prognostic accuracy in both training and validation cohorts for patients with tumors, showing significantly longer survival times for those categorized in the low-risk group compared to the high-risk group. Additionally, our analyses, including tumor mutation burden, immune function, Gene Ontology, Kyoto Encyclopedia of Genes and Genomes pathway enrichment, and drug sensitivity, further elucidated the potential mechanisms through which cuproptosis-associated lncRNAs may influence disease outcome.

**Conclusions:**

The model developed using cuproptosis-related long non-coding RNAs (lncRNAs) demonstrates promising predictive capabilities for both the prognosis and immunotherapy outcomes of tumor patients. This could play a crucial role in patient management and the optimization of immunotherapeutic strategies, offering valuable insights for future research.

**Supplementary Information:**

The online version contains supplementary material available at 10.1186/s12876-024-03219-6.

## Introduction

Hepatocellular carcinoma (HCC) constitutes around 90% of all primary liver cancer instances and ranks as the second leading cause of cancer-related mortality worldwide, with an annual incidence of 850,000 new cases. The principal risk factors for HCC development are well-documented, including infection by hepatitis B and C viruses, alcohol consumption, and exposure to aflatoxin, a fungal metabolite and a major carcinogen globally [1]. There are five main treatment modalities proven to improve the life expectancy of HCC patients: surgical resection, liver transplantation, radiofrequency ablation, chemoembolization, and targeted therapy with the small molecule drug sorafenib [2]. With advancements and the widespread adoption of gene sequencing technologies, research has increasingly focused on the molecular level, aiming to uncover the mechanisms of cancer initiation and progression genetically. Currently, tumor mutation burden (TMB), immune function, and drug sensitivity are at the forefront of hepatocyte research [3].

Long noncoding RNAs (lncRNAs) are a category of noncoding RNA molecules exceeding 200 nucleotides in length, characterized by their lack of open reading frames (ORFs) and absence of protein-coding potential [4]. These molecules play a crucial role in a myriad of cellular processes, including cell differentiation, lineage specification, organogenesis, and tissue homeostasis. Moreover, lncRNAs are implicated in the etiology and progression of various pathological conditions, such as cancer and cardiovascular diseases, thereby emerging as novel biomarkers and therapeutic targets [5, 6]. In the context of HCC, 74 dysregulated lncRNAs have been identified, with 52 exhibiting upregulated expression patterns [7]. Extensive research has demonstrated a significant association between numerous dysregulated lncRNAs in HCC and various clinicopathological features, including characteristics of the primary tumor (size, focality, differentiation, and encapsulation), invasion and metastasis, disease staging, survival rates, and non-tumorigenic aspects like cirrhosis, serum alpha-fetoprotein (AFP) levels, and hepatitis B virus (HBV) infection status [8–10]. This burgeoning interest in lncRNAs as gene transcription products underscores their potential significance in medical research and treatment strategies.

Cuproptosis represents a novel mechanism of cell death, primarily driven by the accumulation of intracellular copper ions. These ions directly interact with the lipoylated components of the tricarboxylic acid (TCA) cycle, leading to the aggregation and malfunction of these proteins, thereby obstructing the TCA cycle. This disruption triggers proteotoxic stress, ultimately culminating in cell death [11]. The balance of intracellular copper is meticulously controlled to ensure copper homeostasis at the cellular level. This regulation involves a sophisticated network of copper-dependent proteins, encompassing copper enzymes, copper chaperones, and membrane transport proteins. These components work in concert to manage copper influx, efflux, and utilization within the cell, ensuring that copper levels remain within a narrowly defined range to maintain copper homeostasis [12]. Copper’s pivotal role in cell signaling underscores its involvement in cancer development and progression, including promoting cell proliferation, angiogenesis, and metastasis [13].

**Fig. 1:**
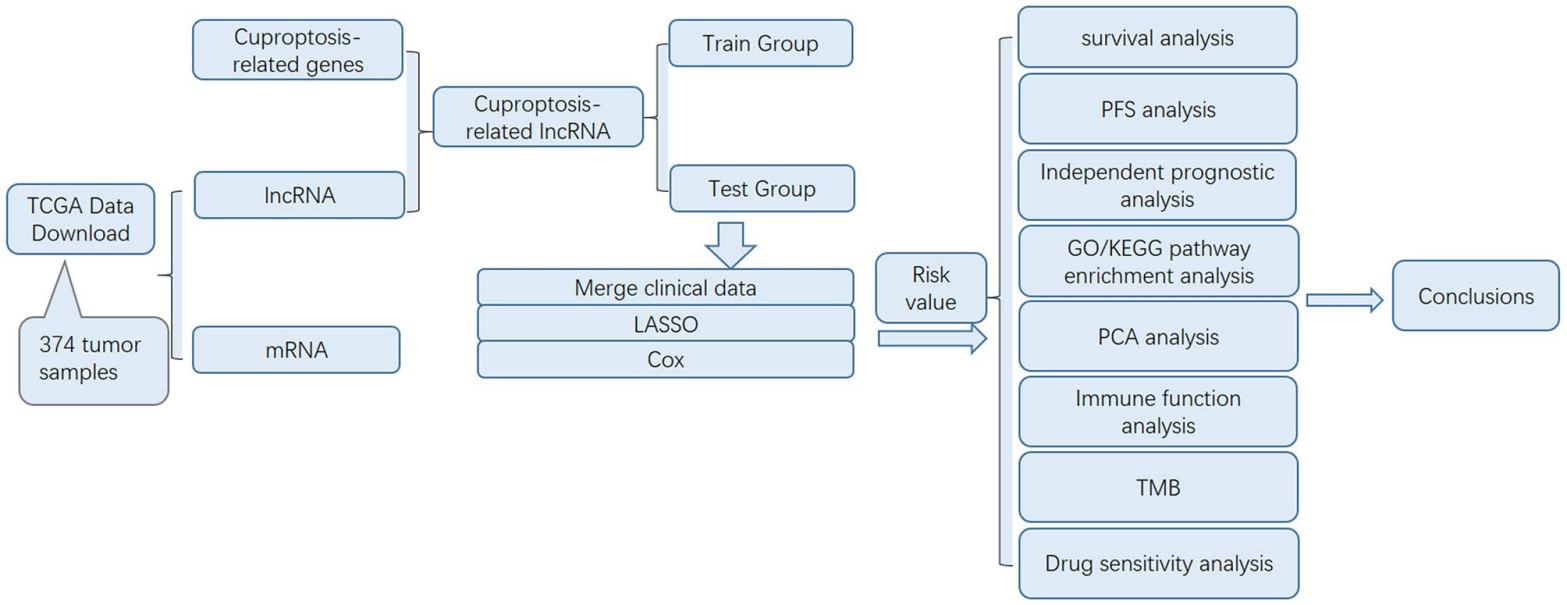
Flow chart of the entire study The figure shows the sources of our data, as well as the main methods of analysis

In this study, we developed a prognostic model based on lncRNAs by identifying cuproptosis-related lncRNAs through the TCGA database and integrating them with the clinical data of patients from the same database. Furthermore, we conducted analyses to correlate the model’s outcomes with mutation profiles, immune functions, and drug sensitivity responses in HCC patients. Pathway enrichment analysis was also carried out to uncover potential underlying mechanisms. The comprehensive methodology and results of our study are illustrated in Fig. 1.

## Methods

### Data acquisition and organization

RNA sequencing data for HCC and the clinical characteristics of patients were sourced from the TCGA database, encompassing 374 HCC tumor samples. We segregated the gene expression matrices into mRNA and lncRNA categories, utilizing the clinical data and lncRNA samples as the foundation for further analysis. Subsequently, we identified 19 genes associated with cuproptosis from existing literature and pinpointed cuproptosis-related lncRNAs via co-expression analysis, laying the groundwork for the development of a prognostic model. The data processing steps included downloading raw data, annotating probes, imputing missing values, and eliminating batch effects. This meticulous processing was undertaken by two experienced bioinformatics analysts. For the analysis, we employed the R software version 4.2.2, making extensive use of the “limma” package [14].

### Prognostic model development

For the construction of the prognostic model, we allocated the HCC tumor samples into a training set and a validation set. The training set underwent LASSO regression analysis utilizing the “glmnet” package [15] to identify more representative genes. Subsequently, univariate Cox regression analysis, facilitated by the “survival” package [16], was employed to select potential prognostic genes. Genes demonstrating statistical significance (p-value < 0.05) in the Cox analysis were flagged as potential prognostic markers. Within the training cohort, patients were stratified into low-risk and high-risk categories based on the median risk score, serving as the division threshold. The prognostic model, established from the training set, was then applied to the validation set samples. This step calculated each sample’s risk score in the validation cohort and assessed the model’s predictive accuracy.

### Correlation analysis

To elucidate the associations between the lncRNAs featured in our model and cuproptosis-related genes, we performed a correlation analysis between these genes and the lncRNAs utilized to construct the model. This analysis aimed to clarify the relationships among different lncRNAs and cuproptosis genes, enhancing our understanding of the interconnectedness between these lncRNAs and the relevant pathways involved in copper metabolism. The threshold for statistical significance was set at *p* < 0.05.

### Survival analysis

We employed Kaplan-Meier curves, using the “survival” package, to illustrate the relationship between survival time and rate among the samples. To delve deeper into the survival differences between high-risk and low-risk groups, we conducted ROC analysis with the “timeROC” package [17] to evaluate the prognostic potential of the genetic markers. Furthermore, to validate our model’s prediction accuracy, we assessed its performance across different subgroups. This involved categorizing samples by gender and disease stage, followed by separate survival analyses for each subgroup. Subsequently, we performed a Progression-Free Survival (PFS) analysis. By integrating clinical data from pan-cancer studies in the TCGA database with the risk scores derived from our model, we divided the samples into high and low groups based on the median risk score. This allowed us to compare the survival differences in progression-free survival status between these two groups, further substantiating the prognostic efficacy of our model.

### Independent prognostic analysis

We conducted both univariate and multivariate prognostic analyses on variables such as age, gender, staging, and grading of the samples, alongside the risk score derived from our model (based on 6 cuproptosis-related lncRNAs), utilizing the “survival” package. This was undertaken to further evaluate whether our constructed models possess the capability to independently predict the prognosis of the samples, distinct from other contributing factors.

### Principal component analysis (PCA)

We executed PCA on the entire gene dataset obtained from TCGA, including all genes, 19 cuproptosis-related genes, cuproptosis-related lncRNAs, and specifically the cuproptosis-related lncRNAs utilized in our model construction (AC026412.3, AC125437.1, AL353572.4, MKLN1-AS, TMCC1-AS1, SLC6A1-AS1). This analysis was conducted using the “limma” and “scatterplot3d” packages [18]. The primary aim was to further validate the capability of our model’s genes to distinguish between samples in high-risk and low-risk groups effectively.

### Immune-related functional analysis

By utilizing the “GSVA” and “GSEABase” packages [19, 20] for immune-related functional analysis, we were able to identify distinct immune-related functions between the high- and low-risk groups. This identification furnishes a valuable reference for further research endeavors.

### Functional enrichment analysis

Integrating the risk scores of each sample with the gene expression matrix, we conducted a functional enrichment analysis on the genes differentially expressed between the high and low-risk groups. Utilizing the “org.Hs.eg.db” and “enrichplot” packages [21, 22], we performed GO/KEGG functional enrichment analysis. Based on the pathways exhibiting differential expression between these groups, we established specific filtering criteria. Subsequently, we focused on pathways that showed pronounced differences in expression between the high and low-expression groups as the subjects for the next phase of our research.

### Analysis of TMB differences

Utilizing the TMB data from 368 samples in the TCGA database, in conjunction with the risk scores assigned to these samples, we analyzed the variations in mutation load between the high- and low-risk groups. Additionally, we examined the mutation differences in the genes included in our model across these risk groups. This approach further elucidated the underlying mechanisms of tumor mutations and their potential impact on risk stratification.

### Drug sensitivity analysis

Leveraging the drug sensitivity data available in the database (https://osf.io/c6tfx/files/osfstorage), we evaluated the sensitivity of each sample to 197 drugs. By integrating these sensitivity scores with the risk scores of each sample, we conducted an analysis to determine the differential drug sensitivities between the high- and low-risk groups. This analysis was performed using the “oncoPredict” package [23], aiming to identify potential therapeutic targets and optimize treatment strategies based on risk stratification.

### Statistical analysis

Data analysis was conducted using R version 4.2.2, with results presented as mean ± standard deviation (SD). Statistical evaluations were performed with SPSS software version 26.0 (SPSS Inc., USA). GraphPad software (version 8.0.2) was utilized for the creation and statistical analysis of graphs. A P-value of less than 0.05 was considered statistically significant.

## Results

### Cuproptosis-related genes and lncRNA co-expression analysis results

**Fig. 2:**
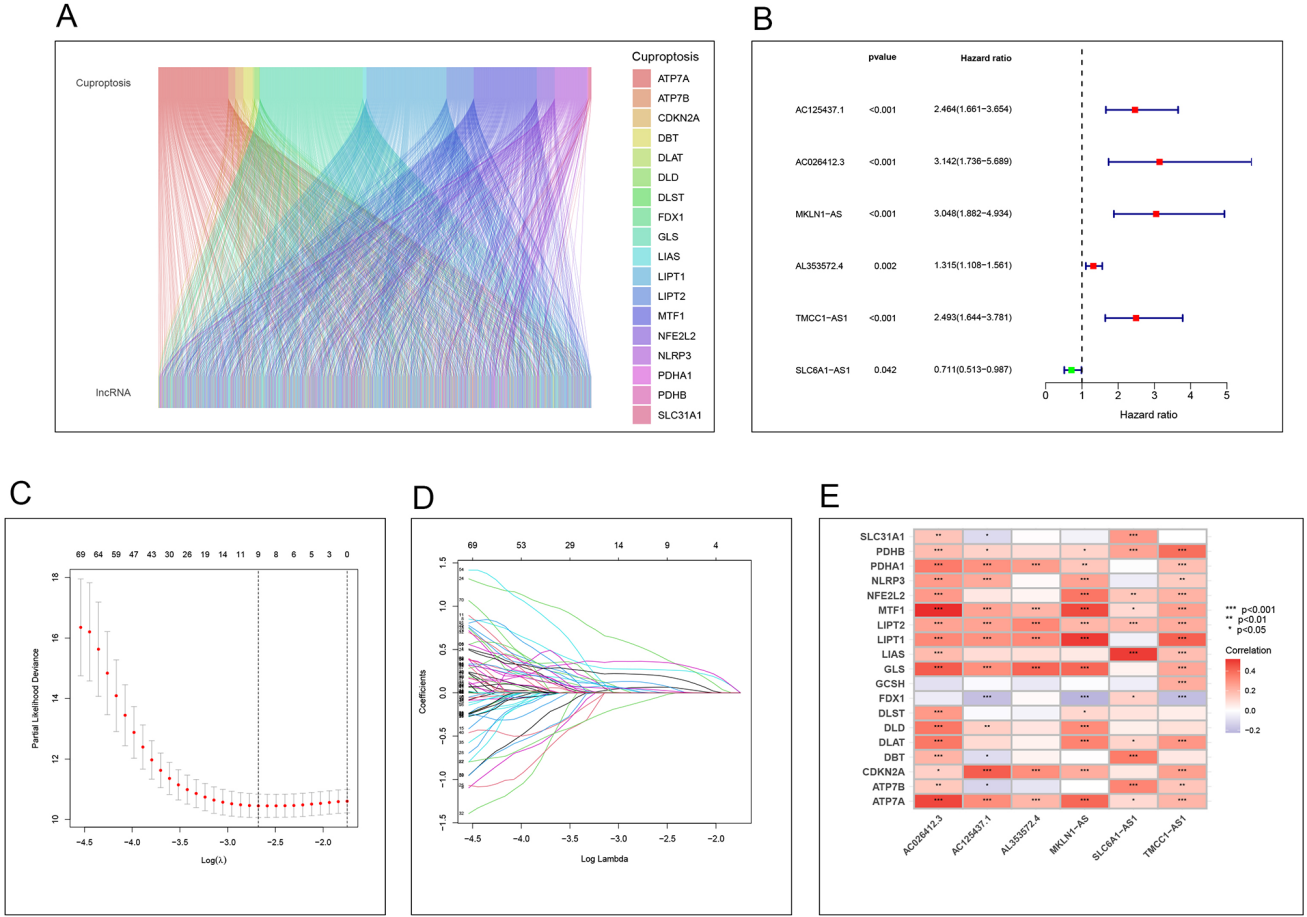
The result of Correlation analysis, Cox regression analysis, and Lasso regression analysis **A** The correlation analysis results between copper death and lncRNA indicate that there are connections between different modules, and different colors represent different copper death-related genes. **B** The result of Cox regression analysis, The red dot represents high risk, while the green dot represents low risk. **C**-**D**: The result of Lasso regression analysis, It can be seen that modeling 6 genes is more accurate and reliable. E: Correlation analysis between the lncRNAs in the model and the Cuproptosis-related genes, Red represents a positive correlation, while blue represents a negative correlation. “*”: *p* < 0.05, “**”: *p* < 0.01, “***”: *p* < 0.001

Through co-expression analysis, we identified 994 lncRNAs associated with cuproptosis. We visualized the interactions between cuproptosis genes and these lncRNAs using a Sankey diagram, with the findings presented in Fig. 2A.

### Prognostic model development

The tumor samples sourced from the TCGA database were partitioned into a training set (185 samples) and a validation set (185 samples). Within the training cohort, univariate Cox regression analysis was conducted to identify potential prognostic genes, with these initial findings illustrated in Fig. 2B. Subsequent LASSO regression analysis was employed to pinpoint more representative genes, the outcomes of which are depicted in Fig. 2C-D. Ultimately, we derived a prognostic model based on 6 cuproptosis-associated lncRNAs: AC026412.3, AC125437.1, AL353572.4, MKLN1-AS, TMCC1-AS1, and SLC6A1-AS1. Utilizing this model, we calculated the risk score for each sample, subsequently dividing the patients into low-risk and high-risk groups according to the median risk score as the threshold.

### Correlation analysis

By conducting a correlation analysis between cuproptosis-related genes and the lncRNAs utilized in our model’s construction, we elucidated the relationships between various lncRNAs and cuproptosis-related genes. This analysis revealed that the cuproptosis-related genes most strongly associated with AC026412.3 were MTF1 and ATP7A; for AC125437.1, the strongest associations were with CDK1 and ATP7A; GLS was the most strongly correlated cuproptosis-related gene with AL353572.4; LIPT1 was identified as the strongest correlate for MKLN1-AS; LIAS showed the strongest association with SLC6A1-AS1; and for TMCC1-AS1, the most significant correlations were with PDHB and LIPT1. These findings are presented in Fig. 2E, offering insights into the specific interactions between cuproptosis-related genes and the lncRNAs in our prognostic model.

### Survival analysis

**Fig. 3:**
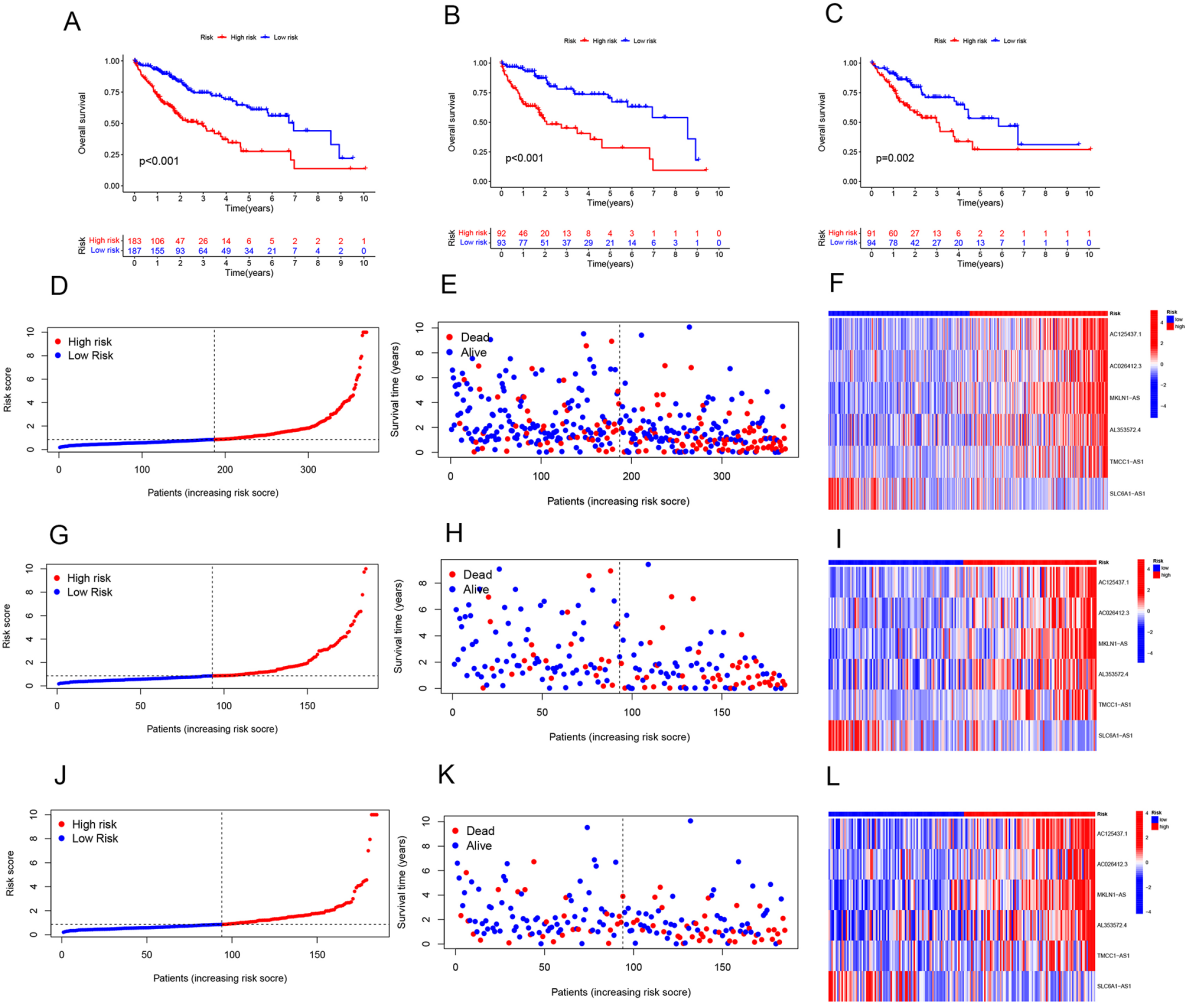
The result of Survival analysis and Risk curve **A** The survival curves of all samples, **B** the survival curve of the training set, **C** the survival curve of the validation set; horizontal coordinates indicate survival time, vertical coordinates indicate survival rate. **D**-**F** risk curve, sample distribution map, and heat map for all sample sets, **G**-**I** risk curve, sample distribution map, and heat map for training sets, **J**-**L** risk curve, sample distribution map, and heat map for validation sets. Red dots indicate low risk or the sample is dead, blue dots indicate high risk or the sample is alive *p* < 0.05

**Fig. 4:**
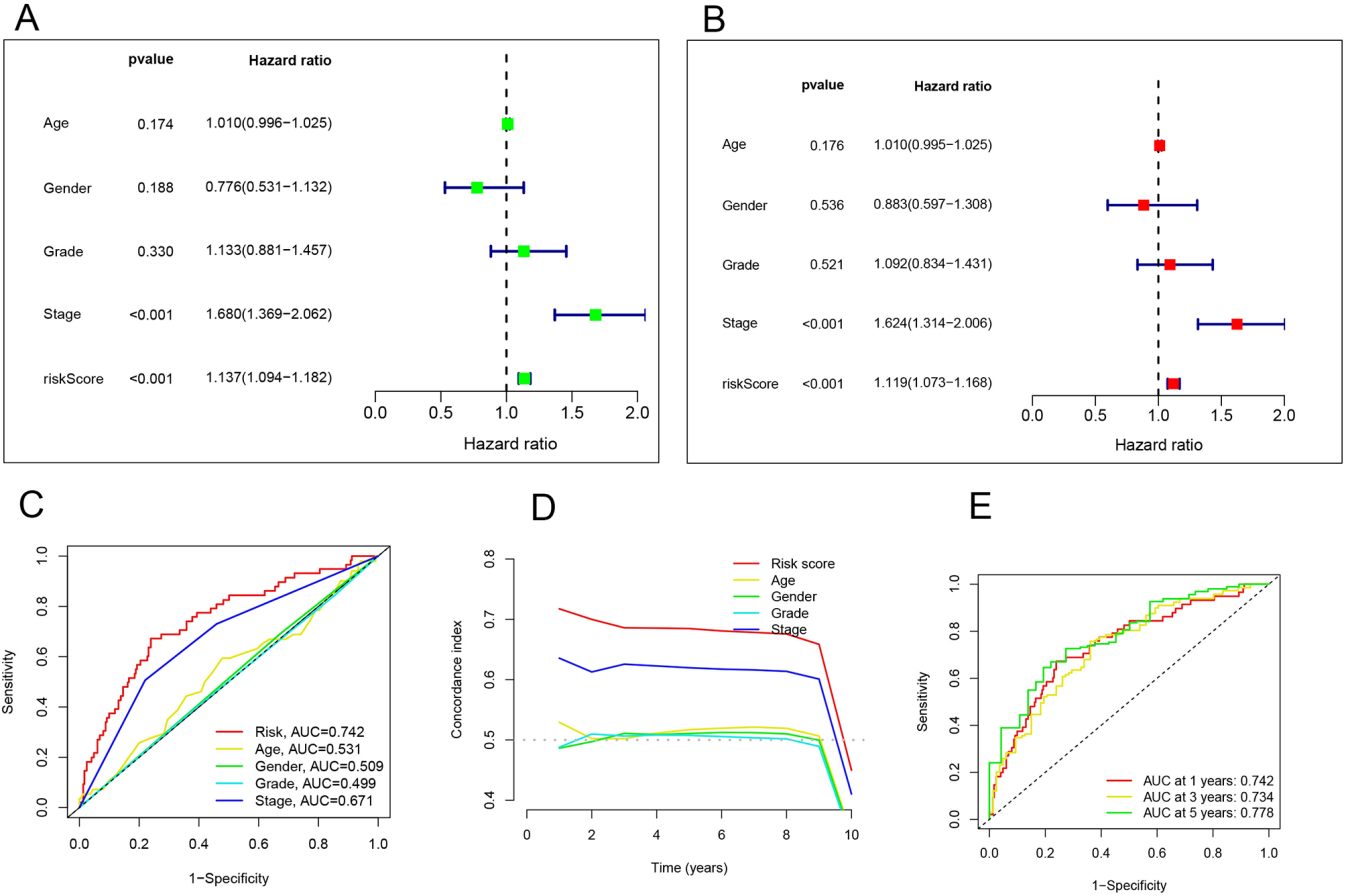
The result of Independent prognostic analysis, C-index curve, and ROC curve **A** Single-factor prognostic analysis. **B** Multivariate prognostic analysis results. Hazard ratio > 1 indicates high factor. **C** ROC curve, **D** C-index curve, Red represents risk score, The larger the area under the curve, the greater the credibility of the results. Risk score is the most accurate prediction result, *p* < 0.05. **E** ROC curve of predicted the survival of the patients at 1, 3, and 5 years. *p* < 0.05

Our survival analysis demonstrated that, across all samples, as well as within the training and validation sets, the survival rate of the low-risk group exceeded that of the high-risk group over time. These outcomes are depicted in Fig. 3A-C. Additionally, we employed risk curves for each sample, revealing that patient mortality in the high-risk group increased over time compared to the low-risk group. Moreover, the expression levels of AC026412.3, AC125437.1, AL353572.4, MKLN1-AS, and TMCC1-AS1 were elevated in the high-risk group, whereas SLC6A1-AS1 showed higher expression in the low-risk group, as illustrated in Fig. 3D-L. To further validate the predictive accuracy of our model, we conducted independent prognostic analyses by categorizing samples based on age, gender, stage, and grade. Our model’s risk scores demonstrated robust predictive capabilities in both univariate and multivariate prognostic analyses, with a P value of less than 0.01, as shown in Fig. 4A-B. Additionally, we assessed the model’s accuracy across different subgroups using ROC and C-index curves for varying ages, genders, stages, and grades, with these findings presented in Fig. 4C-D. We also evaluated the predictive performance of our model for 1-year, 3-year, and 5-year survival rates using ROC curves. The area under the curve (AUC) values were 0.742 at 1 year, 0.734 at 3 years, and 0.778 at 5 years, respectively, underscoring the model’s predictive accuracy at these time intervals, as displayed in Fig. 4E.

### Column line graph for survival prediction

**Fig. 5:**
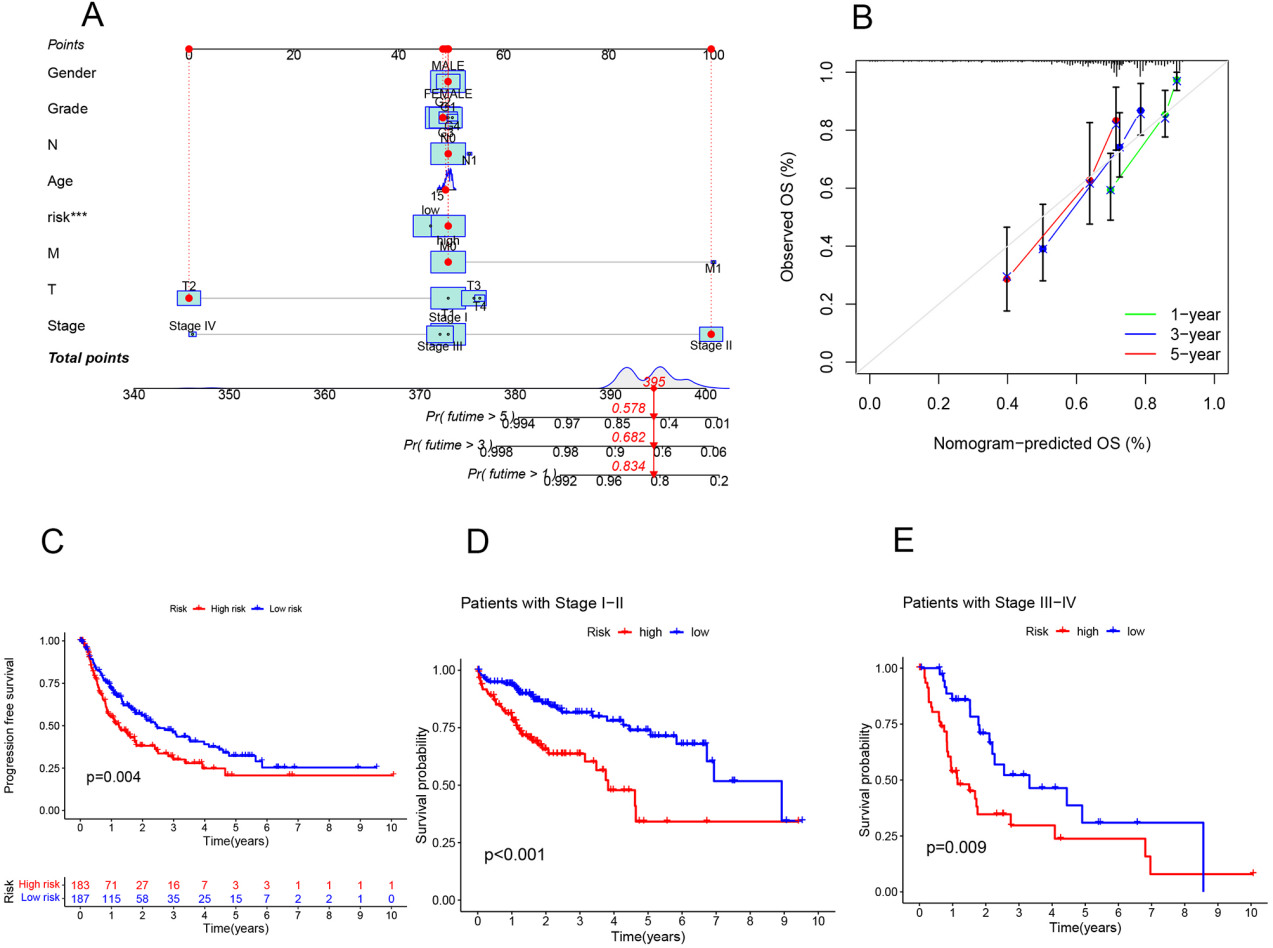
The result of the Column line graph to predict the survival of the sample and survival curve **A** Column line graph to predict the survival of the sample, Predict the 1-year, 3-year, and 5-year survival rates of patients through scoring, using the correction curve of the **B** column chart. **C** The result of PFS analysis, red represents high risk, and blue represents low risk. There is a significant difference in survival time between the high and low risk groups in progression-free survival, *p* < 0.05. **D** The survival curve of early patients, E: The survival curve of late patients, and the survival rate of the low-risk group is higher than that of the high-risk group, *p* < 0.05

We assessed patients based on various criteria, including age, gender, stage, grade, T, N, M classifications, and risk score, to predict their 1-year, 3-year, and 5-year survival rates. This prediction was facilitated through a combined scoring approach, further refined using a calibration curve for accuracy. The outcomes of this analysis are presented in Fig. 5A-B, showcasing the predictive capability of our model for patient survival over these time intervals.

### PFS analysis

Utilizing clinical data from pan-cancer studies in the TCGA database, along with the risk scores derived from our model, we categorized the samples into high and low groups based on their median risk values. We then compared the PFS differences between these groups. Our analysis revealed a significant distinction in PFS between the high-risk and low-risk groups, underscoring the effectiveness of our model in stratifying patients based on their prognosis. These findings are illustrated in Fig. 5C, demonstrating the predictive power of our model in identifying differences in progression-free survival outcomes.

### Survival analysis by clinical staging

We stratified patients into early-stage (Stage I-II) and late-stage (Stage III-IV) groups based on their clinical staging. The survival curves for both groups demonstrated a significant difference in survival between the high-risk and low-risk groups within our model, applicable to both early and late-stage patients. These findings are depicted in Fig. 5D-E, highlighting the model’s capability to differentiate survival outcomes across various stages of disease progression.

### PCA analysis

**Fig. 6:**
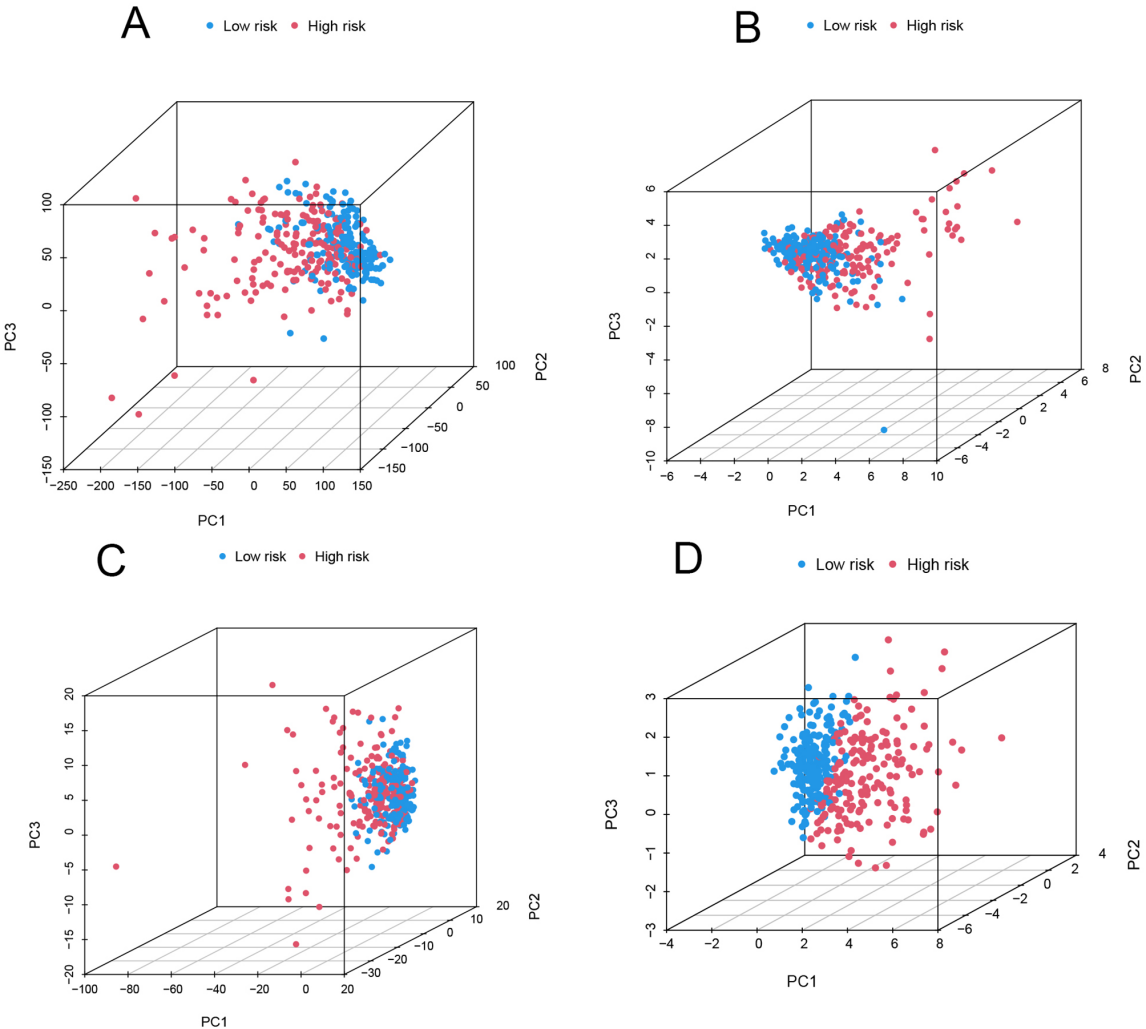
The result of Principal component analysis PCA **A** All genes, **B** Cuproptosis-related genes, **C** Cuproptosis-related lncRNAs, **D** lncRNAs involved in model construction. The red dot represents high risk, while the green dot represents low risk. It can be seen that the lncRNAs involved in the model have the most significant effect in dividing high and low risk groups

The PCA analysis was conducted to assess if the genes utilized in our model could effectively distinguish between high and low-risk groups. This analysis encompassed all genes downloaded from TCGA, 19 cuproptosis-related genes, cuproptosis-related lncRNAs, and specifically the cuproptosis-related lncRNAs incorporated into our model (AC026412.3, AC125437.1, AL353572.4, MKLN1-AS, TMCC1-AS1, SLC6A1-AS1). The results, illustrated in Fig. 6A-D, demonstrate the model’s capability to differentiate between the samples across these categories, highlighting the distinct genetic landscapes of high and low-risk groups.

#### Immune-related function analysis

**Fig. 7:**
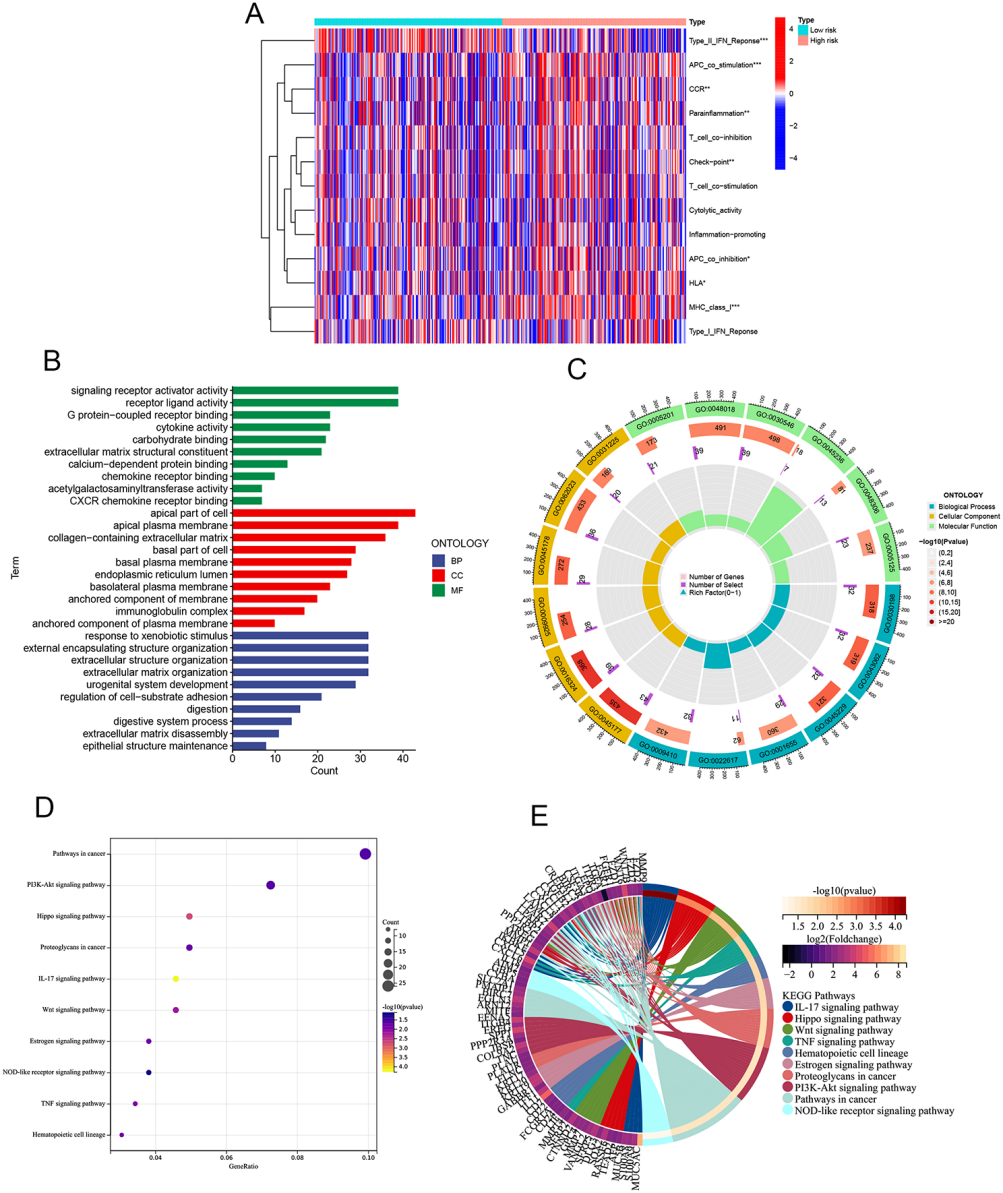
The result of the Analysis of immune-related functions and Functional enrichment analysis **A**: The result of the Analysis of immune-related functions, the red represents high risk, while the green represents low risk. “*”: *p* < 0.05, “**”: *p* < 0.01, “***”: *p* < 0.001, **B**-**C**: The result of GO enrichment analysis, **B** Blue for BP, Red for CC, Green for MF, **C** From outside to inside, the first circle represents the ID of the GO, the second circle represents the number of genes on each GO, the color of the second circle represents the significance of the enrichment, the redder the color means the more significant the enrichment, the third circle represents the number of co-expressed genes, and the fourth circle represents the heat ratio of genes. **D**-**E** The result of KEGG enrichment analysis, The color of the bar graph represents the P-value, the color change from light to dark means that the P-value becomes larger gradually, and the size of the endpoints represents the number of genes enriched in the pathway, the larger the endpoints the greater the number of enriched genes

Through our analysis of immune-related functions, we identified significant differences between high and low-risk groups in specific immune functions, including Type II IFN Response, APC co-stimulation, Parainflammation, Check-point, APC co-inhibition, HLA, and MHC class I. These distinctions highlight the varying immune landscapes present in different risk groups. The results of this analysis are displayed in Fig. 7A, offering insight into the immune mechanisms that may underlie the prognostic differences observed between the high and low-risk groups.

### Functional enrichment analysis

Our functional enrichment analysis unveiled distinct pathways significantly enriched in the high-risk group. As illustrated in Fig. 7B-C, the GO enrichment analysis highlighted key pathways, such as response to xenobiotic stimulus, external encapsulating structure organization, extracellular structure organization, extracellular matrix organization, urogenital system development, regulation of cell substrate adhesion, digestion, digestive system process, extracellular matrix disassembly, and epithelial structure maintenance (BP); anchored component of the plasma membrane, immunoglobulin complex, anchored component of the membrane, basolateral plasma membrane, endoplasmic reticulum lumen, basal plasma membrane, basal part of the cell, collagen-containing extracellular matrix, apical plasma membrane, and apical part of the cell (CC); CXCR chemokine receptor binding, acetylgalactosaminyltransferase activity, chemokine receptor binding, calcium-dependent protein binding, extracellular matrix structural constituent, carbohydrate binding, cytokine activity, G protein-coupled receptor binding, receptor ligand activity, and signaling receptor activator activity (MF). The KEGG enrichment analysis identified pathways more significantly enriched in the high-risk group, including the IL-17 signaling pathway, Hippo signaling pathway, Wnt signaling pathway, TNF signaling pathway, hematopoietic cell lineage, estrogen signaling pathway, proteoglycans in cancer, PI3K-Akt signaling pathway, pathways in cancer, and NOD-like receptor signaling pathway, as shown in Fig. 7D-E. Based on the differential pathway expression between the high and low-risk groups and after establishing filtering criteria, the pathways that exhibited more pronounced differences in expression were selected for further investigation. This strategic approach allows for a focused examination of the mechanisms potentially contributing to the observed prognostic differences.

#### Differential analysis of TMB

**Fig. 8:**
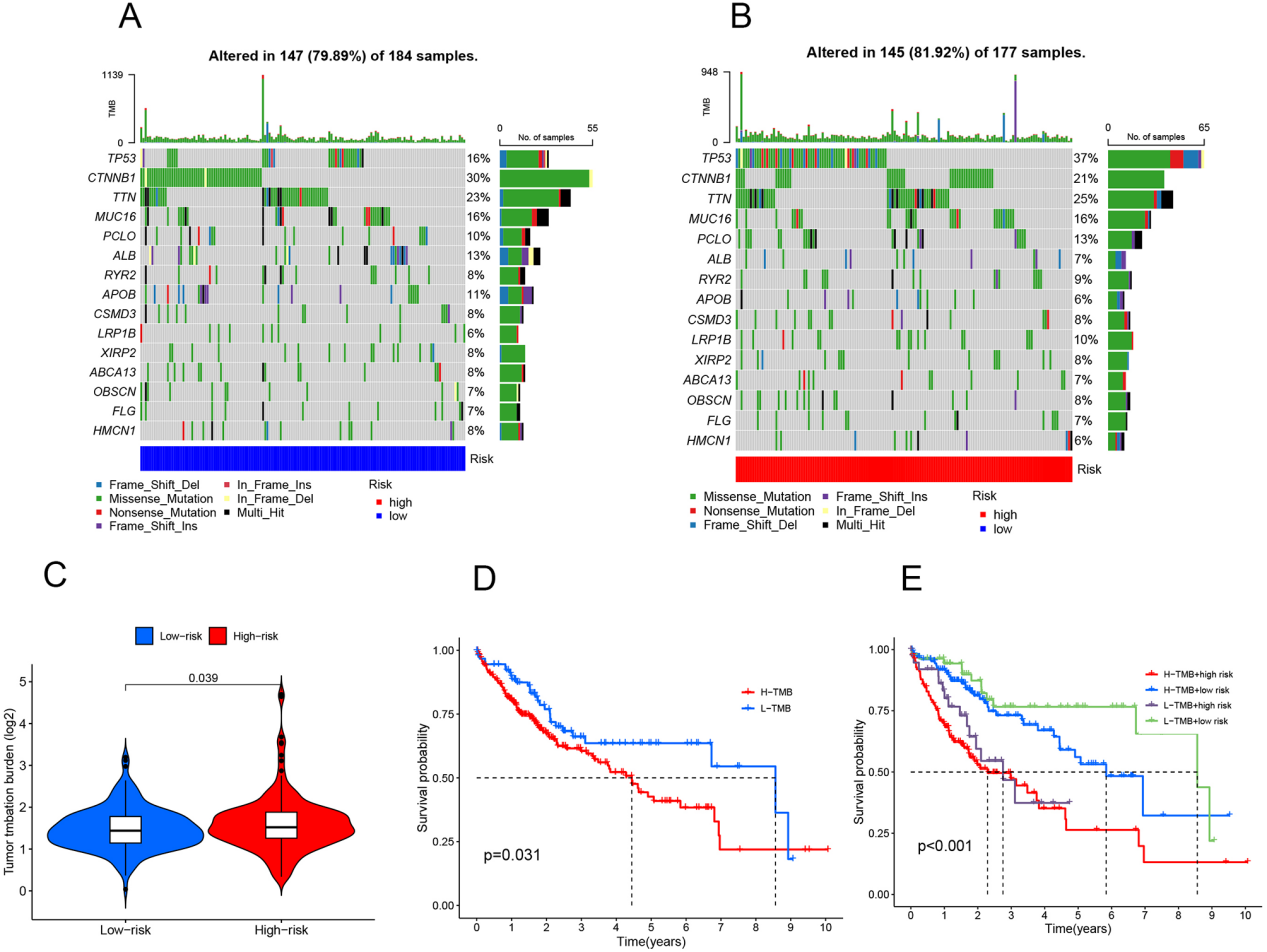
The result of tumor mutation load and survival curve **A**-**B** The waterfall plot of tumor mutations shows the low-risk group in blue and the high-risk group in red. **C** Violin diagram for differential analysis of tumor mutations. Blue represents the low-risk group, while red represents the high-risk group. **D** Survival analysis of high and low mutation groups, blue represents the low mutation group, red represents the high mutation group, and E: Survival analysis of tumor mutation combined with high and low risk groups. Four groups: H-TMB + high risk, H-TMB + low risk, L-TMB + high risk, L -TMB + low risk. *p* < 0.05

Utilizing TMB data from the TCGA database and correlating it with the samples’ risk scores, we depicted the tumor mutation load using a waterfall plot. In the high-risk group, the most frequently mutated genes were TP53 (37%), CTNNB1 (21%), and TTN (25%). Conversely, in the low-risk group, the predominant mutations were found in CTNNB1 (30%), TTN (23%), and TP53 (16%). These observations are presented in Fig. 8A-B. Further analysis of mutation load differences between high and low-risk groups, along with the mutation disparities in genes used in our model, revealed a higher mutation frequency in the high-risk group compared to the low-risk group, as illustrated in Fig. 8C. Incorporating survival data into our analysis, we observed that the survival rate decreases over time in the high mutation group compared to the low mutation group. To enhance the precision of our model, we integrated previous model risk values, categorizing the samples into four groups: High-TMB + High-Risk, High-TMB + Low-Risk, Low-TMB + High-Risk, and Low-TMB + Low-Risk. The survival analysis across these groups highlighted differences in survival rates between the high and low-risk groups within both high and low mutation categories, further validating our model’s effectiveness. These results are showcased in Fig. 8D-E.

#### Drug sensitivity analysis

**Fig. 9:**
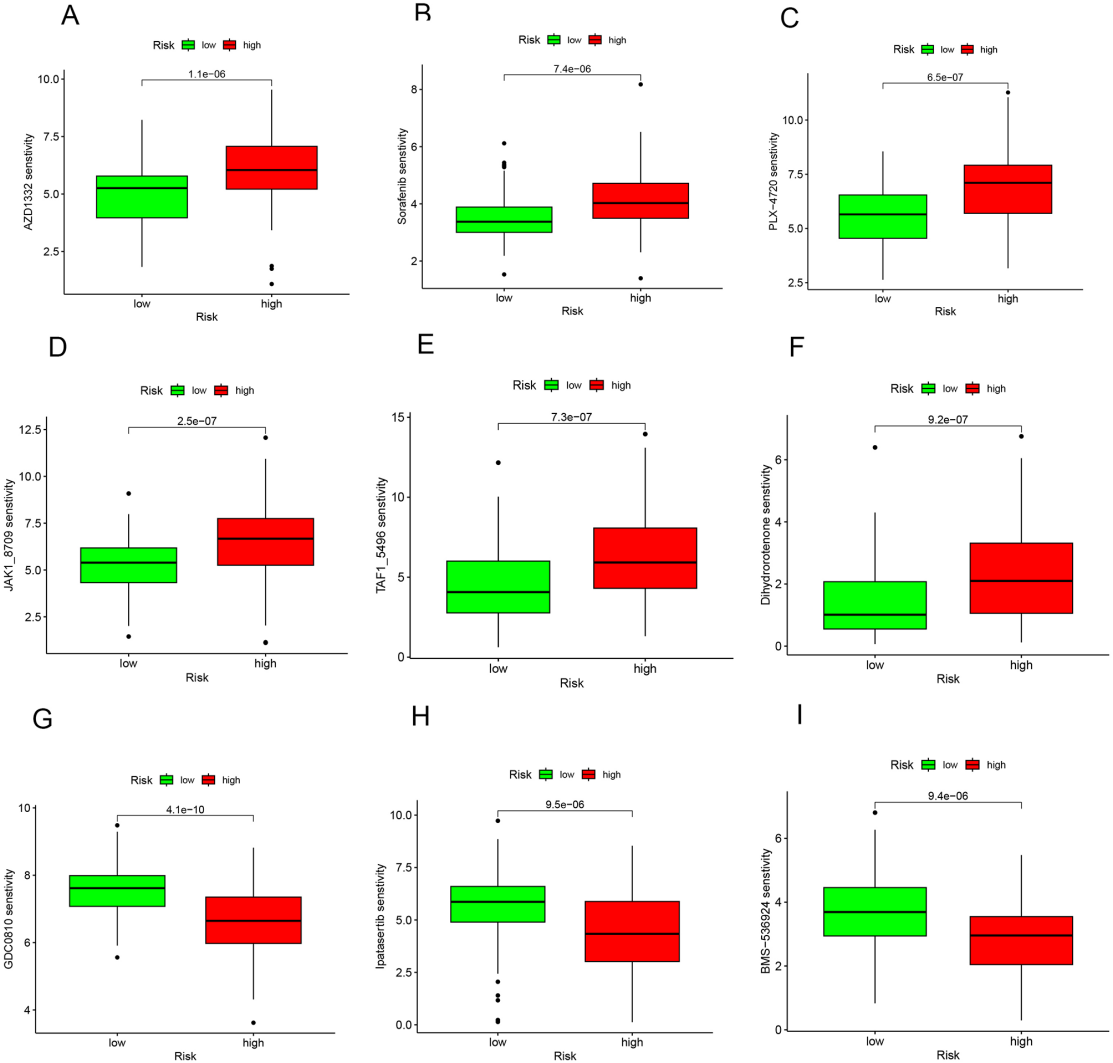
The results of drug sensitivity analysis The horizontal coordinate represents the risk of the sample, with red indicating high risk and green indicating low risk, and the vertical coordinate indicates sensitivity to the drug. *p* < 0.05

Integrating the drug sensitivity data from the database, we assessed each sample’s drug sensitivity and then correlated these scores with the risk values of each sample. This analysis aimed to discern the differences in drug sensitivity between high and low-risk groups. By filtering out non-discriminatory results, we identified disparities in the response to 9 drugs between these groups. These findings, which highlight the variance in drug sensitivity across high and low-risk groups, are illustrated in Fig. 9A-I, providing insights into potential therapeutic targets and treatment optimization based on risk stratification.

## Discussion

Liver cancer remains a significant global health challenge, with HCC ranking as a leading cause of cancer-related mortality worldwide, a trend that continues to escalate. Among all primary liver cancers, HCC is the predominant form of hepatic malignancy. Over the last decade, advances in understanding the molecular pathogenesis of HCC have been substantial. Genomic analyses have delineated the principal drivers of tumor initiation and progression [24], The development of HCC has been attributed to various mechanisms, including gene mutations and immune-related functions [25]. TMB, which quantifies the number of mutations within tumor cells per million base pairs, serves as a critical indicator. A high TMB suggests a multitude of mutations, potentially leading to the generation of novel antigens that can activate the immune response [26]. TMB levels in HCC patients can significantly vary, indicating diverse disease profiles.

Immunotherapy, particularly when involving specific vascular growth inhibitors, has demonstrated significant antitumor efficacy in certain HCC patient subsets. The combination therapy of atezolizumab, an anti-PDL1 antibody, and bevacizumab, a VEGF-neutralizing antibody, is becoming the frontline treatment for HCC [27, 28]. Immune checkpoint inhibitors (ICIs), which boost the immune system’s capacity to attack cancer cells by blocking immune checkpoint proteins like PD-1 and CTLA-4, have achieved remarkable success across various cancer types, including melanoma, lung, and gastric cancers [29]. For HCC treatment, hormilizumab (Nivolumab) and pembrolizumab (Pembrolizumab) are two approved ICIs for advanced stages, significantly enhancing the survival prospects for patients, especially those unresponsive to prior therapies. Despite their success, not all patients exhibit favorable responses to ICIs, prompting ongoing research into predictive markers of treatment response, with TMB emerging as a promising candidate. Recent studies have increasingly focused on genetic mutations and the non-apoptotic cell death pathways in HCC development. The relationship between tumor cell progression, therapy, and the immune microenvironment is intricate. This environment comprises immune cells surrounding the tumor, cytokines, antigen presentation, and immune checkpoint expression, gaining prominence in the diagnosis and treatment strategies for breast cancer [30]. Understanding the dynamics of the immune microenvironment is essential for predicting therapeutic outcomes and devising novel treatments. In HCC, this environment is often modulated by the tumor’s immune evasion tactics, allowing the cancer to suppress immune cell activity and avoid immune detection, thereby facilitating tumor growth and dissemination. However, emerging therapeutic strategies aim to modify the HCC immune microenvironment to enhance susceptibility to immune attacks. Studies have shown that ICI treatment can improve the immune microenvironment in HCC, augmenting T-cell infiltration and promoting anti-tumor immune responses, underscoring the rationale behind immunotherapy’s efficacy [31, 32].

Copper toxicity, a distinct form of copper-dependent cell death, differs from traditional forms of cell death. It is believed that copper directly influences multiple signaling pathways in tumor cells by binding to and activating essential molecules within these pathways [33]. Elevated serum copper ion levels have been observed in patients with lung, prostate, breast, gallbladder, and stomach cancers compared to healthy individuals, highlighting copper’s potential role in tumorigenesis [34–37]. Mutations and dysregulation of LncRNAs are increasingly recognized for their significant impact on cancer [38, 39]. LncRNAs, functioning as both tumor suppressors and oncogenes, have garnered attention as potential novel biomarkers and therapeutic targets due to their widespread and tissue-specific expression patterns [40]. LncRNAs, longer than 200 nucleotides, are integral in regulating chromatin dynamics, gene expression, and cellular processes such as growth, differentiation, and development [41]. They have been shown to influence key pathways in tumor development across various cancers, including leukemia, breast and prostate cancers, lung cancer, and HCC, underscoring their pivotal role in oncogenesis [42, 43]. The involvement of LncRNAs in regulating cancer cell energy metabolism, thus impacting cellular homeostasis and leading to cell death, is a growing area of interest [39, 44]. These cuproptosis-associated LncRNAs may regulate HCC progression by affecting copper ion homeostasis and oxidative stress, potentially inducing cuproptosis and influencing HCC’s pathophysiological processes [45]. The exploration of cuproptosis-associated LncRNAs offers new avenues for HCC treatment, especially as HCC shows considerable resistance to conventional therapies [46, 47], Targeting these LncRNAs could modulate cuproptosis, affecting HCC cell survival and dissemination. Furthermore, these LncRNAs might serve as predictive and diagnostic biomarkers, aiding in early HCC detection and correlating with patient prognosis to guide treatment decisions [48]. Understanding the expression of cuproptosis-associated LncRNAs can assist in tailoring personalized treatment plans, potentially enhancing therapeutic outcomes and patient survival. Recent studies, including this one, have shed light on the roles of cuproptosis and LncRNAs in HCC, focusing on prognosis, diagnosis, immunotherapy, and drug sensitivity [49–58]. This study leverages the novel concept of cuproptosis and its related genes in correlation with LncRNAs to establish a prognostic model for HCC using cuproptosis-associated LncRNAs. Through tumor mutation TMB and functional enrichment analyses, we aim to elucidate the potential mechanisms of these LncRNAs, offering valuable insights for future HCC research and treatment strategies. Investigating cuproptosis-related LncRNAs further can deepen our understanding of HCC pathogenesis and unveil new clinical treatment targets, paving the way for innovative therapies to improve the survival rates and quality of life for HCC patients.

## Conclusions

In this research, we developed a prognostic model using lncRNAs by integrating transcriptome data from HCC cases in the TCGA database with cuproptosis-related genes. We selected lncRNAs associated with cuproptosis as the foundation for our study, further enriching our analysis with clinical data from the database. Our investigation explored the correlation between the model’s outcomes and various factors such as tumor mutation burden, immune function, and drug sensitivity in HCC patients. The findings reveal that the constructed model exhibits robust predictive capabilities, offering valuable insights for future research.

### Electronic supplementary material

Below is the link to the electronic supplementary material.

Supplementary Material 1

Supplementary Material 2

Supplementary Material 3

Supplementary Material 4

## Data Availability

The original data comes from TCGA (https://portal.gdc.cancer.gov/), and the data is accurate.

## Abbreviations

HCC: hepatocellular carcinoma
LncRNA: long non-coding RNA
TCGA: The Cancer Genome Atlas
TMB: tumor mutation burden
GO: Gene Ontology analysis
KEGG: Kyoto Encyclopedia of Genes and Genomes (KEGG) pathway analysis
PFS: Progression-free survival
PCA: Principal component analysis
ICIs: Immune checkpoint inhibitors
