# Supplementary figures and images for: Prognostic analysis of hepatocellular carcinoma based on cuproptosis -associated lncRNAs
Source: BMC Gastroenterol. 2024 Apr 23;24:142. doi: 10.1186/s12876-024-03219-6 (PMC11040954; doi:10.1186/s12876-024-03219-6)

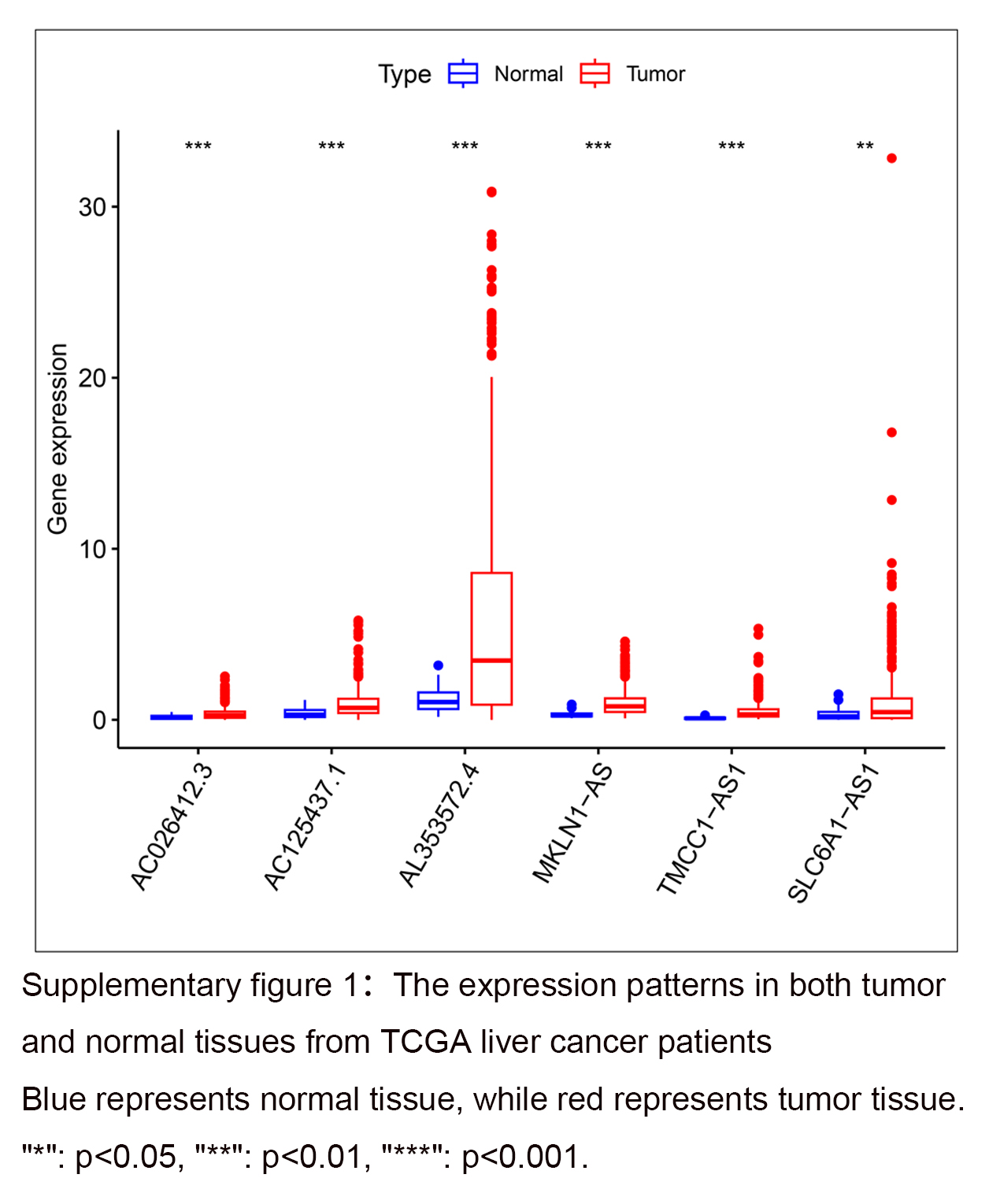

Supplement: Supplementary file 4 — Supplementary Material 4 [file 12876_2024_3219_MOESM4_ESM.jpg]
